# Facts Relative to the Origin of Intermittent Fevers

**Published:** 1797

**Authors:** Thomas Beddoes


II. *Facts relative to the Origin of intermittent Fevers. Communicated in a Letter to Dr.* Simmons, *by* Thomas Beddoes, *M. D.*

I DO not know whether it is the prevailing opinion among medical philosophers, that Dr. Cullen was mistaken in referring the origin of intermittent fevers to effluvia from marshes exclusively. Many physicians believe that they may be excited by other causes; and of this there seems to me to exist in different books such satisfactory evidence, that I should not consider the following cases as worth recording, if the circumstances had not been somewhat peculiar, and at the same time perfectly distinct.

On February 2, 1795, I ordered the first medicines for Miss Sweeting. On the 25th of January, in the evening, she had been chilled in going home; she was unwell the next day, and for several days preceding the 2d of February, had had a daily cold paroxysm, followed by heat and copious perspiration.

I saw her one day in the cold stage of her paroxysm, and it was impossible to imagine more distinct diftinct fhuddering and fhaking. In the interval fhe was lively, and had no complaint; but fhe was beginning to lofe her healthy look. Her complaint was eafily removed; on the 18th of February, however, fhe was threatened with a relapfe, and on the 19th had a flight paroxyfm; but her former medicines at once put an end to the difeafe.

She lived in Rodney Place, and had not been in the valley for a long time. She was at Briftol juft a week before the evening fhe was chilled, but in the intermediate time had never once been off Clifton Hill.

February 4, I was defired to fee Mr. Thomafon; he lived at the Hotwells, near the river. I found him in the hot ftage of his paroxyfm; this was fucceeded by a profufe diaphorefis, and that by a complete intermiffion. The bark freely taken, with fome additions, prevented any return of his fever. About a week before I faw him, Mr. T. had been making fome magnetical experiments in a large cold room, during which he had felt uncomfortably chilled; the following day he fhivered and felt feverifh at times, and he had not been free from thefe feelings for twenty-four hours together, till his complaint was cured by the medicines I have mentioned.

Mrs. —— had clofely attended a fick relation at the Hotwells. In the beginning of February fhe changed her lodgings; the room in which fhe fat the firft day after her removal had been lately wafhed, and was ftill damp; fhe felt indifpofed before bed-time. On the morning of the third day I faw her, fhe had been chilly, was now in the hot ftage of a febrile paroxyfm, and afterwards fell into an exceffive perfpiration; fhe had then a perfect intermiffion, and took medicines fimilar to thofe above mentioned. She feemed recovering as ufual, when fhe imprudently took a cathartic medicine, which operated with great violence. Her fever now returned; it affumed the form of a tertian, and proved very obftinate.

In thefe cafes, where the diforder came on during a fevere froft, it is impoffible to think of marfh miafmata. Indeed, the feelings and obfervations of each patient fo clearly point out the time and manner in which the diforder took place, that I prefume no doubt can remain when both circumftances are confidered.

Opportunities

Opportunities of making obfervations in phyfic, fuperior to all cavil, occur fo feldom, that thought the prefent ought not to be neglected.

*Clifton,*

*March* 7, 1795.

