# Errata in Vol. VII

**Published:** 1800

**Authors:** 


ERRATA IN VOL. VII.

Page 29, line 3, *for* "thought" *read* "I thought."

